# Attempts of Physical Refining of Sterol-Rich Sunflower Press Oil to Obtain Minimally Processed Edible Oil

**DOI:** 10.3390/foods10081901

**Published:** 2021-08-16

**Authors:** Aída García-González, Joaquín Velasco, Leonardo Velasco, M. Victoria Ruiz-Méndez

**Affiliations:** 1 Instituto de la Grasa, Consejo Superior de Investigaciones Científicas (CSIC), Campus/Bd 46, Ctra. de Utrera km 1, E-41013 Sevilla, Spain; aida.garcia@csic.es (A.G.-G.); jvelasco@ig.csic.es (J.V.); 2 Instituto de Agricultura Sostenible, Consejo Superior de Investigaciones Científicas (CSIC), Alameda del Obispo s/n, E-14004 Córdoba, Spain; lvelasco@ias.csic.es

**Keywords:** PS-enriched food, phytosterols, sunflower oil, refined oil, physical refining, chemical refining

## Abstract

New phytosterol (PS)-enriched sunflower seeds, which are higher in campesterol and ∆7-stigmastenol, have recently been developed. Crude oils obtained from these new sunflower seeds in 2015 and 2017 were used in this study. Oils extracted only by press (PO) and with subsequent solvent extraction (SO) were characterized. Physical refining (PhR) was used to obtain edible PO by minimal processing and to keep the PS levels as high as possible. Oils obtained by chemical processing were also studied for comparative purposes. Different bleaching treatments were examined to reduce the contents of phospholipids in the PO to levels required for PhR (<10 mg kg^−1^). Phosphorous levels in PO from 2015 (9–12 mg kg^−1^) were reduced to optimal levels by bleaching with 0.1% Trisyl and 1% Tonsil 278 FF. Contrarily, treatments with Trisyl and Tonsil (278 FF or 114 FF) were not sufficient to reduce the higher levels in PO from 2017 (15–36 mg/kg^−1^), thereby they were subjected to chemical refining (ChR). The PhR applied to PO from 2015 did not lead to substantial changes in the composition and total content of PS. In contrast, losses of up to approximately 30% of total PS were found owing to ChR, although the oils preserved their unique PS profiles.

## 1. Introduction

Plant sterols or phytosterols (PS) are compounds with proven health benefits [1,2]. Their richest natural sources are vegetable oils, followed by nuts and legumes [1]. Extracted from plants, free PS are widely used in fortified foods and dietary supplements. In most cases, free PS are extracted using organic solvents that are harmful to human health as well as to the environment [3]. The food industry is currently facing the challenge of obtaining foods enriched with bioactive compounds while applying minimal processing [4,5]. In this context, the production of PS-enriched seeds may be a good option to increase PS in our diet simply through the intake of vegetable oils [6,7,8,9,10].

The industrial production of sunflower seeds and sunflower oils is of great relevance worldwide [11]. In Europe, sunflower oil is the second most produced oleaginous seed oil and accounts for approximately 30% of the whole oil production [12]. Numerous studies on the production and characterization of edible sunflower oils with modified fatty acid compositions have been reported [13,14]. In addition, the development of oils containing modified tocopherols has been the goal of extensive research [15,16]. However, studies related to sunflower oils from seeds enriched in PS are limited [9,10]. In this respect, there are inherent difficulties in obtaining PS-enriched oils as the content of PS in seeds increases rapidly during the first seed growing stages, when the oil content in the seed is still low, and then the increase rate of PS becomes much slower as compared to that of the oil [9,10]. The PS composition can be also changed [17] and this could be of great interest in order to adapt sunflower oils to specific nutritional demands. Recently, new lines of PS-enriched seeds with increased levels of campesterol and Δ7-stigmastenol, respectively, have been developed [9,10].

At the industrial level, sunflower oil production consists of extraction by press (around 70–80% of the total oil is extracted) followed by solvent extraction (hexane) of the remaining oil. Both the press oil (PO) and solvent oil (SO) are blended to produce what is known as crude oil, which must then be refined for edible purposes [13].

Oils obtained by pressing are highly demanded [18,19]. The fact that POs are obtained without the use of organic solvents raises the question of whether it is worth commercializing them separately in sunflower oil production to obtain added-value environmentally friendly oils. However, the composition of minor components differs between POs and SOs [20,21,22]. Specifically, total PS contents in oils from PS-enriched seeds have been reported to range from 2839 to 5284 mg kg^−1^ for POs and from 4849 to 9249 mg kg^−1^ for SOs [23]. Most importantly, it would be not possible to commercialize POs as unrefined or virgin oils due to their high content in phosphorus (P) and metals, beyond the levels allowed by regulations [24]. While chemical refining (ChR) is imperative in SOs because of its high levels of P, the application of a physical refining (PhR) process to POs when the acidity is low could be an alternative in order to preserve PS, which are otherwise significantly lost during chemical neutralization [25,26,27,28,29,30,31,32,33]. 

PhR basically consists of a bleaching step followed by neutralizing deodorization in which the free fatty acids are removed by steam distillation at low pressures (2–6 mbar) and high temperatures (180–270 °C). Compared to ChR, PhR reduces the loss of neutral oil and allows for the recovery of free fatty acids with minimal production of pollutants [34,35]. The reduction of phospholipids to levels below 10 mg kg^−1^ oil is crucial for PhR because phospholipids promote browning during deodorization [34,35,36].

The removal of low contents of P and metal traces can be achieved by applying specific bleaching treatments [37,38,39]. Bleaching is considered an adsorption purification process by which, along with pigments, a wide range of undesirable components are removed prior to deodorization, including oxidation products, trace metals and other contaminants [37,40]. A treatment with amorphous silica has been reported as a dry degumming process, presenting reductions of P higher than 85% and, thus, avoiding wet-degumming [38,41]. Similar reductions have also been observed for iron [41,42,43]. In this respect, bleaching using combinations of earths and silica could be employed to remove the low levels of P found in POs from new PS-enriched sunflower seeds [23], as well as trace metals to meet the levels allowed in the regulation [24].

The objective of this work was to study the feasibility of PhR, bleaching and distillation to obtain edible POs from new PS-enriched sunflower seeds, thus avoiding chemical treatments. Based upon physical procedures only, the whole process would meet two of the most current consumer demands. On the one hand, oils containing high PS contents naturally would be produced by mechanical means and, on the other hand, a minimal refining processing would be required as compared to classical ChR. The oils obtained from two different harvests, 2015 and 2017, which were characterized in a previous report [23], were studied. Different bleaching treatments in combination with amorphous silica were tested to reduce the phospholipid content to the levels required for PhR (P < 10 mg kg^−1^ of oil). For comparative purposes, ChR and SOs were also investigated to weigh up the pros and cons of considering PO and SO separately in terms of PS levels. The effects of the refining processes on components of interest other than PS, such as fatty acid composition and α-tocopherol contents, were also evaluated.

## 2. Materials and Methods

### 2.1. Chemicals

5-α-cholestan-3-ol and tocopherols (α, β, γ, δ) were standards (Purity 99%) supplied by Sigma-Aldrich Corp. (St. Louis, MO, USA). *N*,*O*-Bis(trimethylsilyl) trifluoro acetamide with 1% trimethyl chlorosilane was used as a silanization reagent and purchased from Supelco Inc. (Bellefonte, PA, USA). All other chemicals and reagents were of analytical grade and were acquired from local suppliers. Trisyl^®^ (GRACE and Co., Columbia, MD, USA) and bleaching earths (Tonsil Optimum 278 FF and Tonsil Supreme 114 FF) were supplied by Clariant Iberica Prod. SA (Toledo, Spain).

### 2.2. Oil Samples

The crude oils were those characterized in a previous report [23]. They were obtained by press and subsequent solvent extraction, respectively, from PS-enriched sunflower seeds that were rich in campesterol (CAM) and Δ7-stigmastenol (STIG), respectively, and from seeds with conventional content and composition of PS, referred to as the control (CON) following the operation conditions described in a previous report [23]. The seeds were harvested in 2015 and 2017, respectively.

### 2.3. Physical Refining

#### 2.3.1. Bleaching

Several bleaching tests were carried out, combining silica gel (Trisyl^®^, Grace and co. Columbia, MD, USA) and different amounts of bleaching earth differing in the degree of activation (Table 1). The bleaching earths were bentonites (CAS Number: 70131-50-9): Tonsil optimum 278 FF (pH 5–7, 10% suspension, filtered) and a very highly active adsorbent, Tonsil supreme 114 FF (pH 2.5–3.5, 10% suspension, filtered), both from Sud-Chemie Adsorbents, Inc. (Germany). 

The procedure used was based on the one proposed in Annexed XIII of Regulation (CEE) N°. 2568/91. The oil sample was placed in a flask and heated at 95 °C under stirring and vacuum conditions. When the set temperature was reached, Trisyl^®^ and the bleaching earth were added. The vacuum was restored and stirring at 95 °C was kept for 10 min. Then the oil was filtered with a MilliPore filter under vacuum. Phosphorous and metal removal was used as a control parameter.

#### 2.3.2. Neutralization/Deodorization

A steam distillation process was carried out at 200 °C for 2 h by applying a vacuum of 0.5–1 mmHg. The source of water vapor was supplied with a peristaltic pump. The water was passed through a separator with controlled temperature and finally introduced through a diffuser into a round flask containing the oil (500 g), applying a steam flow of 0.50 mL min^−1^. Then the oil was cooled and using nitrogen we stopped the vacuum. The deodorized oil was filtered with a filter paper and stored at 5 °C until the analyses.

### 2.4. Chemical Refining

#### 2.4.1. Phosphoric Degumming

Phosphorous compounds in the oils were precipitated with phosphoric acid in water (1:1 *w*/*w*) at 0.1% (*w*/*w*) for PO and 0.2% (*w*/*w*) for SO. The oil/water mixture was vigorously stirred at 40 °C in a paddle stirrer for 5 min.

#### 2.4.2. Neutralization

Without separating the gums from the previous stage, sodium hydroxide was added at different concentrations, depending on the acidity level, so as to be sufficient to neutralize the FFA and the mineral acid added, plus a 10% excess for ensuring the formation of soaps. The sodium hydroxide addition was carried out while stirring at 40 °C. The temperature was then raised to 80 °C and held for 10 min. The resulting soaps, along with the phospholipids hydrated in the previous step, were removed by washing and centrifugation. Centrifugation was carried out at 1295× *g* for 10 min.

#### 2.4.3. Washes

The oils obtained in the previous stage were washed at 90 °C using water at 15% (*w*/*w*). Stirring for 10 min was applied. The water was then separated by centrifugation at 1295× *g* for 10 min. Three successive washing operations were applied to remove the soap completely.

#### 2.4.4. Bleaching and Deodorization

The bleaching and deodorization stages were performed as indicated for PhR.

### 2.5. Analytical Methods

#### 2.5.1. Elemental Analysis

The elemental analysis of metal and non-metal traces—As, Ca, Cu, Fe, K, Mg, Na, P, Pb, S and Zn—was determined by Inductively Coupled Plasma-Optical Emission Spectrometry (ICP-OES) on a Varian ICP 720-ES axial configuration device at the Institute of Natural Resources and Agrobiology (CSIC) (Seville, Spain). SRM 1573a (Tomato Leaves) and an interlaboratory sample (SE 2016 IPE-WEPAL) from the University of Wageningen were used as reference materials. 

#### 2.5.2. Analysis of Chlorophylls

Quantitative determination of chlorophylls in the oils was carried out by spectrophotometry at 670 nm according to Pokorný et al. [44]. Measurements were performed in cyclohexane using 0.3 g·mL^−1^ solutions. A Genesys 10UV spectrophotometer was used.

#### 2.5.3. Acidity

Acidity was determined by titration following the ISO Standard Method 660:2020 [45].

#### 2.5.4. Fatty Acid Composition and PS Contents

The fatty acid composition and the levels of PS were determined according to a method developed in our lab [46], based on a transmethylation reaction of the oil sample followed by a SPE fractionation (NH_2_-500 mg). Two fractions comprising the fatty acid methyl esters and the PS, respectively, were obtained and analyzed by gas liquid chromatography (GLC) [46].

For analysis of fatty acid composition, an Agilent Technologies (AT) 6890 chromatograph equipped with a split/splitless injector, an AT INNOWAX capillary column (100 m × 0.25 mm i.d., ×0.25 µm film thickness) and a flame ionization detector (FID) was used. Hydrogen at a flow rate of 1.2 mL·min^−1^ was used as carrier gas. The injector temperature was 225 °C and a 40:1 split ratio was applied. The oven temperature was kept at 180 °C for 9 min. Then the temperature was increased to 220 °C at 3 °C·min^−1^ and held for 15 min. The FID temperature was set at 250 °C.

For the analysis of PS, the elution solvent was removed at 60 °C under vacuum in a rotary evaporator. The dried extract was silanized and analyzed by GLC. An AT 7890A chromatograph was used, equipped with a split/splitless injector, a capillary column of fused silica HP-5 (5% diphenyl-94% dimethyl-1%vinylpolysiloxane, 30 m × 0.32 mm i.d. × 0.25 µm film thickness) and an FID. The injector temperature was 275 °C and a 20:1 split ratio was used. The oven temperature was 260 °C (isothermal conditions) and the analysis took 40 min. The detector temperature was 320 °C. Hydrogen was used as a carrier gas at a constant pressure of 15 psi.

#### 2.5.5. Tocopherols

Tocopherols were determined by HPLC with fluorescence detection following the ISO standard method 9936:2016 [47]. The oil samples dissolved in n-heptane (50 mg·mL^−1^) were directly analyzed in an Agilent 1260 Infinity HPLC chromatograph (Agilent Technologies, USA) equipped with a quaternary pump VL (G1311C), a standard autosampler (G1329B), a thermostatted column compartment (TCC) (G1316A) and a fluorescence detector (G1321A). A silica HPLC column (LiChrospher^®^ Si 60, 250 mm × 4 mm i.d., 5 µm particle size) (Merck, Darmstadt, Germany) was used. The volume of the sample analyzed was 20 µL. The temperature of the TCC was set at 25 °C. The separation of tocopherols was performed using n-heptane:isopropanol (99:1, *v*/*v*) with a flow rate of 1 mL·min^−1^. The excitation and emission wavelengths in the detector were 290 nm and 330 nm, respectively. Quantification was done by external calibration using tocopherol standards.

### 2.6. Statistical Analyses

The statistical program SPSS (SPSS Inc., Chicago, IL, USA), version 24.0, was used to evaluate the influence of the refining process, considering significance for a probability greater than 95% (*p* < 0.05). The influence of the refining treatment on the different samples analyzed was evaluated by applying the Student’s *t* test.

## 3. Results and Discussion

### 3.1. Bleaching Assays

The POs obtained in harvests from 2015 and 2017 showed different responses to the bleaching conditions applied. The contents of P in the oils from 2015 (9.3–11.9 mg kg^−1^) [23] were reduced to optimal levels for PhR (<10 mg kg^−1^ oil) by applying a bleaching treatment with a blend of Trysil and Tonsil 278 FF (TR1) (Table 2). In contrast, the relatively higher contents in the POs from 2017 (15.4–36.4 mg kg^−1^) as compared to those from the 2015 harvest, did not allow for appropriate reductions of P when treated under the same conditions.

Since it is known that the effectiveness of P reduction is significantly affected by the composition of earths [48], an attempt was made with one of the oils changing the earth to a more activated one (Tonsil 114 FF) and the proportion of the bleaching agents; however, the P levels obtained were not still sufficiently low (Table 2). As discussed below, this fact could be attributed to P coming from sources other than phospholipids in sunflower crude oils [49,50,51]. For appropriate reductions of P, an acid degumming step seemed to be required, which would allow for reductions to levels higher than 90% [39].

The SO obtained either from the 2015 or 2017 harvests presented elevated contents of P (263–585 mg kg^−1^) [23]; hence, the only type of oil refining applicable for the optimal P reduction for deodorization (<10 mg kg^−1^ oil) was acid degumming [34,35,39]. Thus, SO samples were all refined by ChR, which consisted of acid degumming, free fatty acid chemical neutralization, bleaching and deodorization. P was accordingly removed by degumming and finally by bleaching treatments (Table S1), but the remaining P levels were not still adequate for deodorization. Therefore, different bleaching treatments were also examined in the degummed/neutralized oils using different proportions of Trisyl and Tonsil 114 FF (TR4–TR7). The bleaching conditions referenced as TR4 (Table 1) were those selected for refining POs (2017) and those selected as TR7 for SOs.

Table 3 shows the results for the STIG oils after acid degumming, alkali neutralization and bleaching. The levels of P were appropriately reduced for the PO, but the SO still presented relatively high contents with the treatments applied (33.6 mg·kg^−1^). Nevertheless, both treatments were effective in reducing the chlorophyll contents, showing expected losses of approximately 90% [28,30]. 

Even though the levels of P were still relatively high in the degummed/neutralized/bleached SO, no oil darkening was observed in the deodorization step. Elemental analysis also showed that metal traces decreased significantly after the treatments (Tables S1 and S2). Ca and Mg levels were higher in SO than in PO and both were significantly reduced (Table 3). These results are consistent with the losses of P because Ca and Mg are mostly complexed by phospholipids [35,50]. A significant reduction of Fe levels was also achieved in the bleached oils, showing contents below 3 mg kg^−1^, which was likewise indicative of the high efficiency of the bleaching treatments.

Given that P in the PO from the 2015 harvest was reduced by bleaching to levels below 10 mg kg^−1^, which are optimal for PhR (Table 2), the PO-CAM and PO-STIG samples obtained in the 2015 harvest were the only samples that could be refined by PhR, while those obtained in 2017 had to be refined by ChR.

### 3.2. Physical Refining (PhR)

The crude PO from the 2015 harvest met the specifications of edible virgin sunflower oils established by Codex Alimentarius with the exception of the levels of trace metals such as As, Pb and Cu [23]. In terms of acidity, the low values (<0.3%) found were far below the limit established (≤2), but refining was absolutely necessary to meet the metal specifications. In this regard, the bleaching conditions applied to reduce the P levels were also effective to diminish the contents of metals below the specified limits (Table S1).

The effects of PhR on the bleached oils are given in Table 4. Acidity was lower than 0.2%, thus fulfilling the specifications for edible refined oils (Codex Alimentarius), which denoted that appropriate neutralizing deodorization conditions were applied.

#### 3.2.1. Fatty Acid Composition

The results obtained for the fatty acid composition indicated that the refined oils met the characteristics established for sunflower oils [13]. As expected, no substantial changes were observed between the crude and refined oils. Slight but significant (*p* < 0.05) differences were only detected in the STIG sample. Although trans fatty acids could be formed due to a high temperature [28], they were not detected due to the mild conditions applied.

#### 3.2.2. Phytosterols

No significant differences were found in the total content of PS between the crude and refined oils (Table 4). These results are consistent with the mild conditions applied in the bleaching step, using a non-acid activated earth (278 FF), and in deodorization. According to Verleyen et al. [52], the main loss of PS in PhR takes place during the deodorization step, due to the removal of free PS, and mainly depends on temperature. Consequently, together with the absence of trans fatty acids, the unchanged levels of PS obtained were indicative of the mild temperature conditions applied.

Regarding the composition of PS, no significant changes were observed as a consequence of the PhR process either (Table 4). The results confirmed that the conditions selected for bleaching were not drastic enough to cause changes in PS composition due to the dehydration of the Δ5-sterols or the formation of Δ7-sterols isomers. Such reactions would have required temperatures higher than 100 °C and bleaching earths with high activity [25]. As for the deodorization step, preferential losses due to higher volatility of the Δ5 unsaturated PS, i.e., campesterol, stigmasterol, β-sitosterol and ∆7-avenasterol, would have been expected [27]. However, the results obtained showed that the deodorization conditions were not drastic enough to result in partial losses of PS.

#### 3.2.3. Tocopherols

As found for PS, no significant losses of tocopherols were observed after PhR (Table 4). This fact further supported that the bleaching and deodorization steps were run under mild conditions. Tocopherol losses after PhR as high as 20–25%, occurring basically in the deodorization step, have been reported in other studies [27,28,29,30,31,52]. In such cases, tocopherols were normally removed by steam distillation at a high temperature and concentrated in the distillates [7].

### 3.3. Chemical Refining (ChR)

As outlined above, the deodorization conditions applied did not result in oil darkening, even though the content of P was not reduced to optimal levels for deodorization (<10 mg kg^−1^). Considering that bleaching was effective in terms of reductions of metals, especially Ca and Mg, and that most hydratable phospholipids are removed at the degumming stage, the fact that no darkening was observed during deodorization supports the hypothesis that the color could be attributed to compounds others than phospholipids [49,50,51].

#### 3.3.1. Fatty Acid Composition

No substantial changes in fatty acid composition were observed in PO (2017) and SO as a consequence of ChR (Table 5). Only slight but significant (*p* < 0.05) differences were found, in particular for the major fatty acids (C18:1 and C18:2), and in PO to a greater extent.

#### 3.3.2. Phytosterols

As expected, significant losses of PS were observed after ChR (Table 6), ranging from 13% to 32% in POs and from 19% to 31% in SOs. Similar losses of up to 30% were found by Verhé et al. [26] and Verleyen et al. [52]. Due to the presence of a hydroxyl group, free sterols show a certain affinity to the alkaline aqueous solution, and the physical migration of phytosterols seems to be the main reason for phytosterol losses during ChR [19]. In fact, the higher the soda concentration, the higher the losses found [27,28,32,33]. In addition, some losses of PS have also been found in the bleaching and deodorization steps of different vegetable oils [28,32]. 

Regarding the composition of PS, only slight but significant (*p* < 0.05) differences were found between the crude and refined oils (Table 6). The slight differences detected did not follow a definite pattern. Preferential losses of the more volatile PS, i.e., campesterol, stigmasterol, β-sitosterol and ∆5-Avenasterol, were not observed. These results are consistent with the fact that PS were mainly removed in the neutralization step and, hence, no preferential losses of particular PS took place. Despite of the slight differences observed, the oils kept their unique PS compositions, i.e., an abundance of campesterol (CAM) and of ∆7-stigmastenol (STIG), respectively.

#### 3.3.3. Tocopherols

The tocopherol content also decreased after ChR, although to a lower extent as compared to PS (Table 6). The losses found ranged between 5% and 10%. These results are in agreement with other studies, where losses between 5% and 15% were detected [7,28,29,30,31,32,33]. Although the tocopherol content normally decreases gradually throughout the whole ChR process, it is known that the main cause for tocopherol losses is the deodorization step [7].

### 3.4. Pros and Cons of Considering the PO and SO Separately

In terms of PS contents, it would be of great interest to consider the PO and SO separately when the levels of P in PO are low enough for PhR. Taking into account, on the one hand, that PO constitutes around 70% of the oils extracted [23] and, on the other hand, that PhR may not affect the PS content, and only losses up to 30% can occur as a consequence of ChR, processing PO and SO separately would give rise to two refined oils with both containing higher levels of PS than those in the refined blend. In addition, 70% of the crude oil (PO) would be converted into minimally processed edible oil, and the total oil losses and, in turn, the amount of waste and byproducts, would be considerably reduced. When this is not feasible and only ChR can be applied, the reduction of the PS levels in the crude blended oil due to a dilution effect would result in a refined oil with intermediate levels between those in the refined PO and SO. Another fact to be evaluated if considering PO and SO separately is the adaptation of refining conditions, which could complicate the basic operations, and two separate processing lines may be required.

## 4. Conclusions

The P levels present in the PO obtained from the new PS-enriched sunflower seeds can vary from one season to another and their reduction by bleaching to optimal levels for PhR may result as insufficient. If this is the case, the PO of the new seeds should be refined by ChR, consisted of degumming, neutralization, bleaching and deodorization. By applying optimal PhR conditions, the PO of the new cultivars can preserve their naturally occurring levels of PS and tocopherol.

The PO and SO of the new PS-enriched sunflower seeds can lose up to 30% of total PS as a consequence of ChR but keep the PS profile that differentiates them from common sunflower oils. Even when losses of PS are expected after ChR, the levels found in the refined oils, i.e., 3285–4547 mg kg^−1^ in the PO and 4602–6616 mg kg^−1^ in the SO, can be considered high compared to the established ranges by the food codex (CODEX 210-1999) for crude oils (2400–5000 mg kg^−1^).

This study has been the initial stage of characterization of these new oils, aiming at evaluating the optimized refining procedures to maintain their unique sterol composition. Further studies are required to evaluate whether these new oils may have commercial advantages, mainly based on their nutritional properties.

## Figures and Tables

**Table 1:** Proportions of agents used in the different bleaching treatments.

	Treatment	Trisyl (%)	Tonsil 114 FF (%)	Tonsil 278 FF (%)
Physical refining	TR1	0.1	-	1.0
	TR2	0.1	1.0	-
	TR3	0.2	2.0	-
Chemical refining	TR4	0.2	1.0	-
	TR5	0.3	1.0	-
	TR7	0.2	2.0	-
	TR6	0.2	1.5	-

**Table 2:** Phosphorous content (mg kg^−1^) in the press oil samples after bleaching.

	Bleaching Treatment			Phosphorous (mg/kg)	
Oil Sample	Trisyl (%)	Tonsil 114 FF (%)	Tonsil 278 FF (%)	Initial	Final
2015					
CON	0.1	-	1.0	11.7	4.2
CAM	0.1	-	1.0	9.3	3.7
STIG	0.1	-	1.0	11.9	5.9
2017					
CON	0.1	-	1.0	27.3	24.2
CAM	0.1	-	1.0	15.4	10.7
STIG	0.1	-	1.0	36.4	30.1
STIG	0.1	1.0	-	36.4	28.9
STIG	0.2	2.0	-	36.4	24.4

Abbreviations: Conventional (CON), Δ7-stigmastenol-rich (STIG) and campesterol-rich (CAM) sunflower oils. Results express the mean value of two determinations (*n* = 2).

**Table 3:** Influence of degumming/neutralization/bleaching on the levels of components determined by elemental analysis (mg kg^−1^) and of chlorophyll (mg kg^−1^) in the conventional (CON), Δ7-stigmastenol-rich (STIG) and campesterol-rich (CAM) sunflower oil samples of 2017.

	Bleaching Treatment			Phosphorous (mg/kg)	
Oil Sample	Trisyl (%)	Tonsil 114 FF (%)	Tonsil 278 FF (%)	Initial	Final
2015					
CON	0.1	-	1.0	11.7	4.2
CAM	0.1	-	1.0	9.3	3.7
STIG	0.1	-	1.0	11.9	5.9
2017					
CON	0.1	-	1.0	27.3	24.2
CAM	0.1	-	1.0	15.4	10.7
STIG	0.1	-	1.0	36.4	30.1
STIG	0.1	1.0	-	36.4	28.9
STIG	0.2	2.0	-	36.4	24.4

nd, not detected. Results express the mean value of two determinations (*n* = 2).

**Table 4:** Influence of the physical refining (PhR) process on fatty acid composition (%) and PS levels of the press oils of 2015.

	CAM		STIG	
	Bleached	Ph Refined	Bleached	Ph Refined
Acidity (%)	0.26 ± 0.03a	0.15 ± 0.05b	0.18 ± 0.02a	0.05 ± 0.03b
Fatty acid composition (%)				
C16: 0	7.1 ± 0.03a	7.1 ± 0.05a	6.1 ± 0.02a	6.0 ± 0.06a
C16: 1	nd	nd	nd	nd
C18: 0	2.9 ± 0.03a	3.0 ± 0.10a	4.3 ± 0.03a	4.7 ± 0.11b
C18: 1	34.7 ± 0.15a	35.0 ± 0.68a	39.6 ± 0.15a	40.0 ± 0.18b
C18: 2	54.5 ± 0.12a	54.1 ± 0.68a	49.0 ± 0.10b	48.3 ± 0.21a
C20: 0	0.3 ± 0.02a	0.3 ± 0.01a	0.4 ± 0.00b	0.3 ± 0.03a
C22: 0	0.4 ± 0.02a	0.4 ± 0.04a	0.5 ± 0.00a	0.5 ± 0.01a
C24: 0	0.2 ± 0.01a	0.2 ± 0.01a	0.2 ± 0.01a	0.2 ± 0.02a
Phytosterols Composition (%)				
Campesterol	29.5 ± 0.46a	29.6 ± 0.43a	6.4 ± 0.22a	6.5 ± 0.08a
Stigmasterol	7.5 ± 0.24a	7.4 ± 0.19a	8.8 ± 0.27a	8.4 ± 0.18a
β-Sitosterol	47.9 ± 0.78a	48.3 ± 0.49a	49.3 ± 0.29a	49.6 ± 0.31a
∆5-Avenasterol	3.3 ± 0.12a	3.3 ± 0.11a	1.2 ± 0.09a	1.2 ± 0.04a
∆7-Stigmastenol	7.8 ± 0.69a	7.5 ± 0.48a	29.7 ± 0.42a	30.0 ± 0.28a
∆7-Avenasterol	4.0 ± 0.34a	3.9 ± 0.35a	4.6 ± 0.18a	4.4 ± 0.14a
Total PS content (mg kg^−1^)	3387 ± 145a	3285 ± 56a	4056 ± 53a	3818 ± 232a
Tocopherols (mg·kg^−1^)	881 ± 22a	861 ± 21a	722 ± 18a	702 ± 17a

nd, not detected. Results are expressed as mean values ± standard deviation (*n* = 4 for initial oils and *n* = 3 for refined). Different letters indicate significant differences between the bleached and PhR samples (*p* < 0.05) according to the Student’s *t* test.

**Table 5:** Influence of the chemical refining (ChR) process on the fatty acid composition (%) of the press and solvent oils of 2017.

	CON		CAM		STIG	
	Crude	Ch Refined	Crude	Ch Refined	Crude	Ch Refined
Press	****	****	****	****	****	****
Acidity (%)	1.00 ± 0.05a	0.02 ± 0.01b	0.99 ± 0.05a	0.02 ± 0.01b	1.10 ± 0.05a	0.02 ± 0.01b
Fatty Acids (%)						
C16: 0	7.0 ± 0.01a	7.0 ± 0.01a	6.5 ± 0.01a	6.6 ± 0.00b	7.1 ± 0.01a	7.2 ± 0.01b
C16: 1	0.2 ± 0.00a	0.2 ± 0.00a	nd	0.1 ± 0.00	0.1 ± 0.00a	0.1 ± 0.00a
C18: 0	3.6 ± 0.01b	3.5 ± 0.02a	3.0 ± 0.01b	2.9 ± 0.01a	4.1 ± 0.03a	4.1 ± 0.01a
C18: 1	30.0 ± 0.02a	30.3 ± 0.06b	30.0 ± 0.08a	30.3 ± 0.03b	23.8 ± 0.02a	24.4 ± 0.02b
C18: 2	57.9 ± 0.05b	57.7 ± 0.09a	59.5 ± 0.09b	59.1 ± 0.05a	63.6 ± 0.02b	62.8 ± 0.02a
C20: 0	0.3 ± 0.00a	0.3 ± 0.01a	0.3 ± 0.04b	0.2 ± 0.00a	0.3 ± 0.00a	0.3 ± 0.00a
C18: 3	0.1 ± 0.00a	0.1 ± 0.01a	nd	0.1 ± 0.01	0.1 ± 0.00a	0.1 ± 0.01a
C20: 1	0.2 ± 0.00a	0.1 ± 0.01b	0.1 ± 0.02a	0.2 ± 0.00a	0.2 ± 0.00a	0.2 ± 0.01a
C22: 0	0.6 ± 0.00a	0.6 ± 0.01a	0.4 ± 0.05b	0.5 ± 0.01a	0.6 ± 0.01a	0.6 ± 0.01a
C24: 0	0.3 ± 0.00a	0.3 ± 0.01a	0.1 ± 0.02a	0.1 ± 0.02a	0.3 ± 0.00a	0.3 ± 0.01a
Solvent	****	****	****	****	****	****
Acidity (%)	1.42± 0.05a	0.08± 0.03b	1.64 ± 0.10a	0.10± 0.05b	1.55 ± 0.05a	0.09 ± 0.02b
Fatty Acids (%)						
C16: 0	7.3 ± 0.0b	7.2 ± 0.01a	6.8 ± 0.03a	6.7 ± 0.03a	7.4 ± 0.00a	7.4 ± 0.04a
C16: 1	0.2 ± 0.00a	0.2 ± 0.01a	nd	0.1 ± 0.00a	0.1 ± 0.00a	0.1 ± 0.00a
C18: 0	3.6 ± 0.01b	3.5 ± 0.01a	3.2 ± 0.01a	3.1 ± 0.04a	4.1 ± 0.01a	4.0 ± 0.10a
C18: 1	29.0 ± 0.03a	29.3 ± 0.01b	29.6 ± 0.04b	27.9 ± 0.07a	23.6 ± 0.02a	24.4 ± 0.20b
C18: 2	58.5 ± 0.06a	58.5 ± 0.01a	59.3 ± 0.07a	59.3 ± 0.09a	63.3 ± 0.02	62.7 ± 0.38a
C20: 0	0.3 ± 0.00a	0.3 ± 0.00a	0.2 ± 0.00a	0.2 ± 0.00a	0.3 ± 0.00a	0.3 ± 0.01a
C18: 3	0.1 ± 0.00a	0.1 ± 0.01a	nd	0.1 ± 0.01a	0.1 ± 0.00a	0.1 ± 0.00a
C20: 1	0.2 ± 0.00a	0.2 ± 0.00a	0.2 ± 0.00a	0.2 ± 0.00a	0.2 ± 0.00a	0.2 ± 0.01a
C22: 0	0.6 ± 0.01b	0.5 ± 0.01a	0.5 ± 0.01a	0.5 ± 0.01a	0.6 ± 0.00a	0.6 ± 0.02a
C24: 0	0.3 ± 0.00a	0.3 ± 0.00a	0.2 ± 0.00a	0.2 ± 0.01a	0.3 ± 0.00a	0.3 ± 0.01a

nd, not detected. Results are expressed as mean values ± standard deviation (*n* = 4 for bleached oils and *n* = 3 for ChR oils). Different letters indicate significant differences between bleached and ChR oils for each sample (*p* < 0.05), according to the Student’s *t* test.

**Table 6:** Influence of the ChR process on minor components (PS and tocopherols) of the press and solvent oils of 2017.

	CON		CAM		STIG	
	Crude	Ch Refined	Crude	Ch Refined	Crude	Ch Refined
Press						
Phytosterols composition (%)						
Campesterol	6.2 ± 0.06a	6.1 ± 0.27a	28.2 ± 0.1b	26.5 ± 0.39a	6.2 ± 0.06a	6.5 ± 0.01b
Stigmasterol	7.8 ± 0.08b	7.1 ± 0.33a	8.9 ± 0.29b	7.7 ± 0.19a	9.4 ± 0.19a	9.4 ± 0.17a
β-Sitosterol	61.2 ± 0.44a	62.6 ± 1.17a	47.5 ± 0.07b	46.3 ± 0.74a	46.6 ± 1.1a	48.8 ± 0.16b
∆5-Avenasterol	1.6 ± 0.04	nd	3.7 ± 0.07b	2.6 ± 0.11a	0.9 ± 0.03	nd
∆7-Stigmastenol	18.8 ± 0.51a	20.3 ± 1.03a	6.7 ± 0.18a	12.6 ± 1.24b	32.5 ± 1.58a	30.9 ± 0.33a
∆7-Avenasterol	4.5 ± 0.1b	3.9 ± 0.19a	4.9 ± 0.12b	4.2 ± 0.05a	4.3 ± 0.28a	4.5 ± 0.11a
Total PS content(mg kg^−1^)	4476 ± 136b	3897 ± 225a	5225 ± 203b	3533 ± 63a	5284 ± 200b	4547 ± 298a
Tocopherols (mg kg^−1^)	515 ± 13a	489 ± 12b	881 ± 22a	810 ± 20b	722 ± 18a	679 ± 17b
Solvent						
Phytosterols Composition (%)						
Campesterol	7.3 ± 0.15a	7.2 ± 0.25a	23.4 ± 0.19a	23.2 ± 0.15a	8.3 ± 0.04a	8.5 ± 0.19a
Stigmasterol	11.4 ± 0.14b	10.7 ± 0.15a	11.0 ± 0.11b	10.2 ± 0.17a	12.6 ± 0.25b	11.2 ± 0.08a
β-Sitosterol	57.9 ± 0.2a	58.9 ± 0.38b	50.9 ± 0.35a	52.7 ± 0.58b	48.0 ± 0.09a	48.1 ± 0.26a
∆5-Avenasterol	1.7 ± 0.04	nd	3.3 ± 0.03b	2.5 ± 0.13a	1.5 ± 0.05b	1.2 ± 0.03a
∆7-Stigmastenol	17.3 ± 0.46a	18.8 ± 0.22b	7.7 ± 0.64a	7.6 ± 0.48a	24.8 ± 0.48a	26.4 ± 0.09b
∆7-Avenasterol	4.4 ± 0.19a	4.3 ± 0.27a	3.6 ± 0.05a	3.9 ± 0.18b	4.6 ± 0.22a	4.7 ± 0.14a
Total PS content(mg kg^−1^)	7272 ± 343b	5858 ± 310a	6678 ± 387b	4602 ± 238a	9249 ± 669b	6610 ± 458a
Tocopherols (mg kg^−1^)	517 ± 12a	480 ± 12b	912 ± 23a	871 ± 21b	784 ± 20a	713 ± 17b

nd, not detected. Results for FA and PS are expressed as mean values ± standard deviation (*n* = 4 for crude oils and *n* = 3 for refined oils). Different letters indicate significant differences between crude and refined oils for each sample (*p* < 0.05), according to the Student’s *t* test.

## Supplementary Materials

The following are available online at https://www.mdpi.com/article/10.3390/foods10081901/s1, Table S1: Elemental analysis (mg kg^−1^) (*n* = 2) and chlorophyll content (mg kg^−1^) (*n* = 2) of the press and solvent oils obtained in 2017 after different refining treatments. Table S2: Elemental analysis (mg kg^−1^) (*n* = 2) of the press oils obtained in 2015 after bleaching treatment.

## Abbreviations

Phytosterol	PS
Oil obtained by press	PO
Solvent extracted oil	SO
free fatty acids	FFA
Chemical Refining	ChR
Physical Refining	PhR
phosphorous	P
Sunflower oil rich in campesterol	CAM
Sunflower oil rich in Δ7-stigmastenol	STIG
Conventional sunflower oil	CON
