# Supplementary material for: Attempts of Physical Refining of Sterol-Rich Sunflower Press Oil to Obtain Minimally Processed Edible Oil
Source: Foods. 2021 Aug 16;10(8):1901. doi: 10.3390/foods10081901 (PMC8394065; doi:10.3390/foods10081901)
Supplement: Supplementary file 1 [file foods-10-01901-s001.zip › foods-1341810-Supplementary.pdf]

Table S1.-Elemental analysis (mg kg^{-1}) (n=2) and chlorophyll content (mg kg^{-1}) (n=2) of the press and solvent oils obtained in 2017 after different bleaching treatments that were not effective.

	**Press CON**		**Press CAM**		**Press STIG**				**Solvent STIG**			
	**Initial**	**TR1**	**Initial**	**TR1**	**Initial**	**TR1**	**TR2**	**TR3**	**Initial**	**TR4**	**TR5**	**TR6**
**P**	**27.3**	**24.2**	**15.4**	**10.7**	**36.4**	**30.1**	**28.9**	**24.4**	**585.1**	**62.6**	**39.2**	**47.1**
As	nd	nd	nd	nd	nd	nd	0.1	nd	nd	nd	0.1	nd
Ca	78.5	21.2	58.6	35.6	106.9	39.9	27.5	49.2	305.4	95.5	31.1	40.7
Cu	1.1	0.1	0.2	0.3	1.1	0.2	0.6	0.2	1.1	0.4	0.1	0.2
Fe	6.0	1.3	4.6	1.6	5.8	1.4	0.9	1.8	32.0	10.7	3.9	4.0
K	6.7	3.8	7.3	1.7	6.4	5.2	3.9	2.4	95.4	6.1	3.6	3.4
Mg	11.6	12.2	6.3	7.5	14.4	13.0	11.8	11.3	158.2	19.8	12.7	15.1
Na	4.0	3.6	2.9	4.2	6.1	5.6	3.6	4.9	9.2	14.1	13.2	13.9
Pb	nd	nd	0.2	nd	0.4	nd	nd	0.1	0.4	0.4	0.3	0.1
S	13.0	12.2	6.6	12.3	14.3	11.8	11.3	14.2	15.0	7.3	14.8	14.8
Zn	1.0	0.5	0.6	0.6	1.5	2.6	0.5	0.8	4.6	1.9	0.8	0.8
Chlorophylls	2.7	1.0	1.5	1.1	6.9	3.1	2.5	1.4	21.2	1.6	1.6	1.8

nd, not detected. Coefficient of variation ≤ 7%. See Table 1 for the bleaching agents used.

Table S2.-Elemental analysis (mg kg^{-1}) (n=2) of the press oils obtained in 2015 after bleaching treatment with Trisyl 0.1 % and Tonsil 278 FF 1.0% (TR1).

	CON		CAM		STIG	
	**Initial**	**Final**	**Initial**	**Final**	**Initial**	**Final**
As	0.9	0.5	1.0	nd	0.7	0.1
Ca	31.8	7.0	24.1	8.4	21.9	7.5
Cu	0.4	0.1	0.5	0.1	0.4	0.1
Fe	7.0	2.0	3.1	2.5	4.4	2.1
K	7.6	2.6	7.2	5.1	4.8	4.9
Mg	6.7	4.6	5.8	5.1	4.6	5.4
Na	3.0	22.8	7.6	7.4	nd	10.8
Pb	nd	nd	0.2	0.1	0.1	0.1
S	11.4	10.1	11.6	5.8	7.3	18.2
Zn	4.7	4.6	0.8	0.6	1.4	1.6

nd, not detected. Results express the mean value of two determinations (n=2). Coefficient of variation ≤ 7%.
